# Preparation and Antibacterial Properties of Poly (l-Lactic Acid)-Oriented Microporous Materials

**DOI:** 10.3390/biom14111432

**Published:** 2024-11-11

**Authors:** Yihong Li, Yanjun Feng, Qingyi Huang, Cenyi Luo, Wei Chen, Zhengqiu Li, Lei Liu, Jiafeng Li

**Affiliations:** 1 School of Material Science and Engineering, Xihua University, Chengdu 610039, China; liyihonglq@126.com (Y.L.); huanghuanlzq@126.com (Q.H.); luocenyi@163.com (C.L.); 18780358062@163.com (W.C.); 2 CCTEG Coal Mining Research Institute, Beijing 101399, China; fengyjchn@gmail.com (Y.F.); ljfhngs@126.com (J.L.); 3 State Key Laboratory for Animal Disease Control and Prevention, College of Veterinary Medicine, Lanzhou University, Lanzhou Veterinary Research Institute, Chinese Academy of Agricultural Sciences, Lanzhou 730000, China; shaning0606@126.com

**Keywords:** poly (l-lactic acid), oriented micropores, self-reinforcing, antibacterial properties

## Abstract

In this manuscript, an efficient self-reinforcing technology—solid hot drawing (SHD) technology—was combined with green processing supercritical carbon dioxide (SC-CO_2_) foaming technology to promote poly (l-lactic acid) (PLLA) to form an oriented micropore structure. In addition, Polydimethylsiloxane (PDMS), with a high affinity of CO_2_ and biological safety, was introduced to enhance the nucleation effect in SC-CO_2_ foaming and co-regulate the uniformity of oriented micropores’ structure. The results showed that orientation induced PLLA crystallization, so the tensile strength was improved; the maximum tensile strength of the oriented micropores’ PLLA reached 151.2 MPa. Furthermore, the micropores mainly improved the toughness; the maximum elongation at break reached 148.3%. It is worth mentioning that PDMS can form an antibacterial film on the surface of the material, so that the material has a continuous antibacterial effect.

## 1. Introduction

Compared to traditional conventional packaging materials, poly (l-lactic acid) (PLLA) has good biocompatibility and degradability, but is difficult to prepare into a light and uniform film while ensuring its strength and toughness, which limits its development in the packaging industry. In order to enhance the mechanical strength of PLLA, researchers used a number of methods, including a variety of physical and chemical methods [1,2,3,4,5]. Among these methods, the self-reinforcing method stood out because of its simple, fast and efficient advantages, harnessing self-reinforcing solid hot drawing (SHD) technology to prepare high-strength PLLA-oriented samples, which achieved 200 MPa [5].

However, increasing the tensile strength of PLLA is always accompanied by the sacrifice of toughness; the high crystallization caused by the orientation puts the molecular chain in a frozen state, limiting the movement of the molecular chain. If the molecular chain can be given more space to move while the orientation is enhanced, it is possible to achieve the toughening and strengthening of PLLA at the same time. Therefore, the idea to construct a microporous structure for the oriented PLLA was provided. The purpose of foamed PLLA is mainly to dissolve the physical blowing agent (such as SC-CO_2_ or N_2_) in the PLLA matrix and generate thermodynamic instability under a certain temperature and pressure, resulting in the nucleation and growth of the pore, and then, through rapid pressure relief and other ways, discharge the dissolved gas out of the matrix to obtain a microporous structure [6,7]. The construction of a microporous structure provides more movable space for the molecular chains, and re-mobilizes the movement vitality of the frozen molecular chains, as well as the plasticizing effect of SC-CO_2_ [8,9,10]; the toughness of the oriented PLLA is greatly increased.

Nevertheless, although a microporous structure can greatly enhance the toughness of PLLA, disordered micropores greatly reduce the performance of PLLA; it is still a challenge to prepare PLLA-foamed materials with uniform microporous structures via SC-CO_2_ foaming technology [11,12,13,14,15]. At present, there are several methods to control the porous structure of oriented PLLA: adding chain extenders to obtain a branched structure [6,13,16], regulating the molecular weight of PLLA [12,13,17,18], introducing different types of additives to achieve melt enhancement [19,20,21,22,23,24,25,26], and increasing the crystallinity of PLLA [27,28,29,30]. Yujing Tang et al. obtained branched PLLA by mixing linear PLLA with a chain extender and adding different contents of a self-assembled nucleating agent to further change its crystallization properties and melt enhancement [31]. Compared to pure branched PLLA, the density of PLLA microcellular foam with 1 wt % self-assembly nucleating agent (TMC) was increased by two orders of magnitude and the foaming expansion rate was as high as ten times. Composite modification is still the most simple and effective method. Polydimethylsiloxane (PDMS), the simplest and most common silicone compound, is non-toxic and did not cause physiological reactions in the human body. More importantly, PDMS is also a CO_2_-philic additive, which could be proven to promote micropore nucleation by enhancing the adsorption and dissolution of CO_2_ [32] and increase the density and uniformity of the micropores [33,34,35]. PDMS has been recognized as the material of choice for many emerging technologies, and, as a result, significant advances have been made in functional silicones over the past few years [36,37,38,39,40,41]. However, there are few reports about PDMS as a bio-additive added to PLLA to prepare biodegradable packaging materials [42,43].

In order to simultaneously improve the strength and toughness of PLLA and broaden the application of PLLA in the packaging field, PLLA-oriented microporous materials were constructed by combining SHD technology and SC-CO_2_ foaming technology. Moreover, PDMS was applied to regulate the uniformity of micropores and endow the materials with stronger antibacterial properties, so as to obtain lightweight, high-strength and antibacterial PLLA materials. Furthermore, the interaction mechanism between PDMS and PLLA molecular chains was investigated. The effects of orientation, foaming on the structure and the properties of the material were explained. Finally, based on the adhesion and growth situation of bacteria with material, the antibacterial mechanism of the material was explored.

## 2. Experimental Section

### 2.1. Material

PLLA 3052D was purchased from Nature Works Co., Plymouth, MN, USA. Its density was 1.24 g/cm^3^, with 4% D-lactic; the melt index was 14 g/10 min (210 °C, 2.16 kg). Commercial-purity-grade CO_2_ (99% purity, Chengdu Guoguang Industrial Gas Co., Ltd., Chengdu, China) was used as the physical blowing agent. PDMS was purchased from Thermo Fisher Scientific; the cas number was 9016-00-6, with an average of M_w_~4000.

### 2.2. Preparation of Samples

#### 2.2.1. Preparation of PLLA/PDMS Composites

Before use, the PLLA particles were dried for 4 h (80 °C) until reaching a moisture content below 0.025% (250 ppm). Firstly, the PLLA particles were melted into a twin-screw extruder (Nanjing Shengchi rubber Machinery manufacturing Co. Ltd., SHJ-35, Nan Jing, China) with an average temperature of 185 °C and then cooled for granulation. Secondly, isotropic PLLA samples were obtained via conventional injection molding (HAITIAN MA900IIS/280, Zhejiang, China), with a condition of 185 °C, 15 MPa, and a mold temperature of 50 °C, referred to as PLLA-0. Similarly, after mixing PLLA and 1 wt% PDMS evenly, PLLA/PDMS-0 was also prepared after processing by the twin-screw extruder and injection molding machine.

#### 2.2.2. PLLA and PLLA/PDMS Oriented Materials Were Prepared by SHD Technology

Oriented PLLA with draw ratios of 400% and 800% was prepared using SHD technology. Similarly, oriented PLLA/PDMS with draw ratios of 400%, 800% and 1200% was prepared. The draw rate was 480 mm/min at a temperature of 120 °C. Finally, the oriented samples were cooled to room temperature and then unloaded to maintain the oriented structure. To facilitate this, the oriented PLLA with a 400% draw ratio was labeled PLLA-4, while the oriented PLLA/PDMS with a 400% draw ratio was labeled PLLA/PDMS-4.

#### 2.2.3. Construction of Uniform Cell Materials by SC-CO_2_ Foaming Technology

According to the previous work of the research group, the temperature, pressure and time parameters of SC-CO_2_ batch foaming were determined [5]. The oriented samples were put into a reactor (SLM10, Beijing Century SenLong experimental apparatus Co., Ltd., Beijing, China) (65 °C), injected with CO_2_ using a double-plunger pump (2ZB-2L20A, Beijing Xingda Technology Development Co., Ltd., Beijing, China) at 20 MPa to put the CO_2_ in a supercritical state, immersed with the samples for 30 min, then had their temperature raised to 80 °C (20 MPa), and immersed for 30 min. Then, the pressure was quickly released, and the micropores were formed. To facilitate this, PLLA/PDMS-x-F signified the foam samples, and x referred to the draw ratio of oriented PLLA, such as PLLA/PDMS-4-F.

### 2.3. Measurements

#### 2.3.1. Fourier Infrared Spectroscopy (FTIR)

The transmitted infrared spectrum of the sample was measured by a Fourier infrared spectrometer (VERTEX70, BRUKER, Karlsruhe, Germany). The wave number range was 600 cm^−1^~4000 cm^−1^, and the resolution was 4 cm^−1^.

#### 2.3.2. Differential Scanning Calorimetry (DSC)

The melting behavior of the PLLA samples was characterized using a DSC instrument (TA DSC Q200, Waters Technology Co., Ltd., Shanghai, China) in a nitrogen atmosphere. The non-isothermal DSC assessment was performed by heating 5–10 mg samples from 25 °C to 230 °C at a rate of 10 °C/min. The Xc of the PLLA samples was calculated using Equation (1):

(1) $$Xc=\frac{\left(\Delta Hm-\Delta Hc\right)}{\Delta H{}^{\circ}m}\times 100\%$$

where ΔHm is the melting enthalpy, ΔHc is the cold crystallization enthalpy, and ΔH°m (ΔH°m = 93 J⋅g^−1^) is the ΔHm for 100% crystalline PLLA.

#### 2.3.3. Scanning Electron Microscopy (SEM)

The SEM samples were first cut along the orientation direction of the samples with a knife, and then frozen in liquid nitrogen for more than 1 h. Finally, the samples were broken along a small opening with tweezers and observed after spraying gold. The morphology of oriented microporous samples was obtained via SEM (TESCAN MIRA3, Brno, Czech Republic) at a voltage of 5 kV.

#### 2.3.4. Mechanical Property Measurements

The mechanical properties were measured by a 4302-material testing machine (Electronic universal material testing machine, Instron LTX-850, Boston, MA, USA) according to ISO 527 [44]. The tests were repeated five times to obtain average values (with a speed of 50 mm/min).

#### 2.3.5. Surface Tension Measurements

The contact angle of the pure PLLA sample and modified samples was measured and recorded by a contact angle tester (JH-901A, Jinhuayi Technology Co., Ltd., Beijing, China). The contact angle medium used in the test was distilled water and ethylene glycol and the size of the liquid drop was 15 μL; each group of materials was measured 5 times to obtain the average value.

The surface tension was determined using the Owens–Wendt–Raeble–Kaeble (OWRK) regression model, which calculated the surface tension using known polar components (γ^p^) and dispersive components (γ^d^) of liquids such as water and ethylene glycol [45]. Consequently, the interaction force ($\gamma_{s-\mathrm{soybean}\;\mathrm{oil}}$) between the material and soybean oil was determined based on the polar and dispersive components of soybean oil [46]. The calculation formulas of surface tension and the interaction force between material and oil are shown in Equations (2), (3) and (4), respectively, while the polar and dispersion components of various liquids are shown in Table 1 [45,46].

(2) $$\frac{1+cos\theta }{2}\frac{\gamma_{l}}{\sqrt{\gamma_{l}^{d}}}=\sqrt{\gamma_{s}^{d}}+\sqrt{\gamma_{s}^{p}}\sqrt{\frac{\gamma_{l}^{p}}{\gamma_{l}^{d}}}$$

(3) $$\gamma_{s}=\gamma_{s}^{p}+\gamma_{s}^{d}$$

(4) $$\gamma_{s-\mathrm{soybean}\;\mathrm{oil}}={\left[\sqrt{\gamma_{\mathrm{soybean}\;\mathrm{oil}}^{p}}-\sqrt{\gamma_{s}^{p}}\right]}^{2}+{\left[\sqrt{\gamma_{\mathrm{soybean}\;\mathrm{oil}}^{d}}-\sqrt{\gamma_{s}^{d}}\right]}^{2}$$

where θ is the contact angle of the test liquid, $\gamma_{l}$ is the surface energy of the liquid, and $\gamma_{l}^{p}$ and $\gamma_{l}^{d}$ are polar component and dispersion components of the test liquid.

#### 2.3.6. Antibacterial Property Test

##### Bacterial Biofilm Text

Bacterial biofilm refers to the substance secreted by bacteria on the surface of the material. Bacteria adhere closely to the material through the biofilm, and proliferate and grow on this basis [47,48]. Therefore, the growth and proliferation of bacteria on the material can be reflected by measuring the relative expression of the biofilm. In this manuscript, we used Staphylococcus aureus (a representative of Gram-positive bacteria) and Pseudomonas aeruginosa (a representative of Gram-negative bacteria) for antibacterial experiments and tests.

We performed inoculation with an amount of 10^5^ CFU/mL on different samples’ surfaces, and cultured them in bacterial culture dishes to form bacterial biofilm. After bacterial culture for a period of time (Day 1/Day 3/Day 7/Day 10), the samples were taken and tested. We absorbed and discarded the bacterial solution, dried in a biosafety cabinet for 3 h, dyed each hole with 200 μL crystal violet for 10 min, absorbed and discarded excess crystal violet, left it overnight to dry, added 2 mL ethanol to each hole after dyeing, and then added 25 μL dye solution and 175 μL anhydrous ethanol to the 96-well plate. The test was carried out at 595 nm with an enzyme-labeled instrument. For comparison, we set a blank group (abbreviated as B) in only Luria–Bertani (LB) medium. For the control group (abbreviated as C), without samples, only bacteria were co-cultured with LB.

##### Bacterial Growth Condition

The different morphologies of bacteria on the surface of the materials was captured by SEM (TESCAN MIRA3, Brno, Czech Republic) with the extension of culture time (1 Day/3 Day/10 Day), at a voltage of 5 kV. The energy-dispersive spectrometer (EDS) used surface scan mode at a voltage of 10 kV to obtain the surface element distribution of samples.

##### Interaction Force Between Bacteria and Material Surface

The main components of the membrane of most bacteria include a phospholipid bilayer, and the main components of the phospholipid bilayer are 1,2-dipalmitoyl-sn-glycero-3-phosphocholine(DPPC), 1,2-dioleoyl-sn-glycero-3-phosphocholine(DOPC) and 1-palmitoyl-2-oleoyl-sn-glycero-3-phosphocholine(POPC). POPC is the main component that inhibits its phase separation and maintains its structural microdomain stability [49]. Therefore, the contact angle between POPC, distilled water and glycerol [50] was selected to calculate the dispersion component $\gamma_{POPC}^{d}$ (=46.06 mN/m), polarity component $\gamma_{POPC}^{p}$ (=1.73 mN/m) and total surface energy $\gamma_{POPC}$ (=47.82 mN/m) of POPC by Equations (2) and (3). Thus, a new equation can be derived from Equation (4):

(5) $$\gamma_{s-bacteria}={\left[\sqrt{\gamma_{POPC}^{p}}-\sqrt{\gamma_{s}^{p}}\right]}^{2}+{\left[\sqrt{\gamma_{POPC}^{d}}-\sqrt{\gamma_{s}^{d}}\right]}^{2}$$

### 2.4. Statistical Analysis

All the experiments were conducted in triplicate, and the data were analyzed via one-way analysis of variance (ANOVA and Tukey’s post hoc test) using GraphPad Prism 5.0. Data were presented as means ± SEM. Normality assumptions were checked using quantile–quantile plots, while a data log transformation was performed to correct non-normality prior to the analysis. *p*-values < 0.05 were considered statistically significant.

## 3. Results and Discussion

### 3.1. FTIR

**Figure 1:**
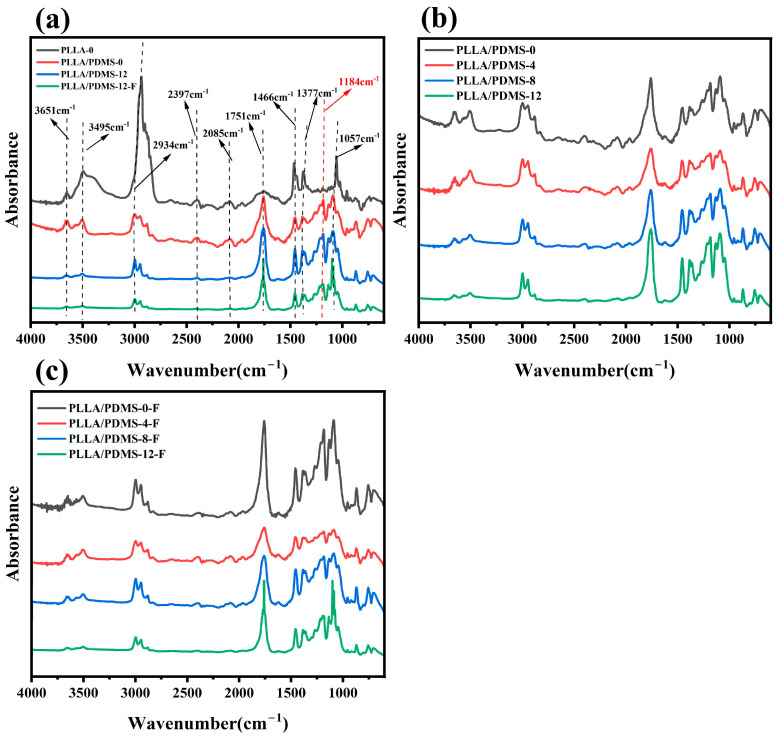
FTIR curves of (**a**) pure PLLA, adding PDMS, stretching, and foaming samples; (**b**) different orientation ratios after adding PDMS; (**c**) different draw ratios of foamed samples.

The FTIR results are shown in Figure 1. In order to eliminate the interference between different samples and compare the difference in peaks between different spectra, the infrared spectra were normalized with C=O peaks.

The FTIR spectra of PDMS-modified PLLA is shown in Figure 1a, with distinct peaks at 1184 cm^−1^ for Si-O stretching [51], 1057 cm^−1^ for C-O-C antisymmetric stretching, 1377 cm^−1^ and 1466 cm^−1^ for C-H bending in methyl groups, and 1751 cm^−1^ for C=O, indicative of esters [52,53]. The CO_2_ peak at 2397 cm^−1^ reflected PLLA’s interaction with atmospheric CO_2_ [54]. As shown in Figure 1b, orientation slightly affected the main chain but significantly enhanced the C=O peak and weakened C-H vibrations on the side chain, suggesting the orientation’s greater impact on side chains. The CO_2_ signal diminished due to molecular chain rearrangement. Figure 1c shows that foaming enhanced the C-O-C peak and reduced Si-O and C-H vibrations, indicating increased chain mobility. Foaming also eliminated CO_2_, as the gas escaped during rapid pressure relief, leaving a reduced residual CO_2_. Overall, foaming and orientation had a more pronounced effect on PLLA’s side chains than the main chain.

**Figure 2:**
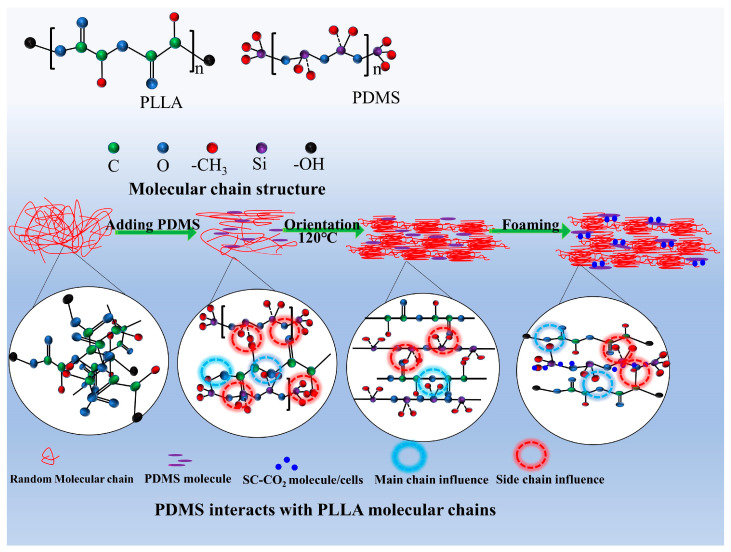
The mechanism of PDMS affecting the movement of PLLA molecular chains during orientation and foaming.

The above data illustrate the mechanism of PDMS affecting the molecular chain activity of PLLA, as shown in Figure 2. The addition of PDMS made the PLLA molecular chain have certain kinetic activity, and the interaction between the main chain and the side chain groups of PLLA was strengthened. Orientation further improved the motility of PLLA’s side chain because the main chain’s movement was limited. As for the foamed samples, due to the plasticizing effect of SC-CO_2_ and there being more movement space for molecular chains, the movement vitality of the main chain and side chains of the molecules after orientation were reactivated, so PLLA still had a good movement ability under the high-strength structure of the orderly arrangement; this was key to realizing the toughening and strengthening of PLLA.

### 3.2. DSC

**Figure 3:**
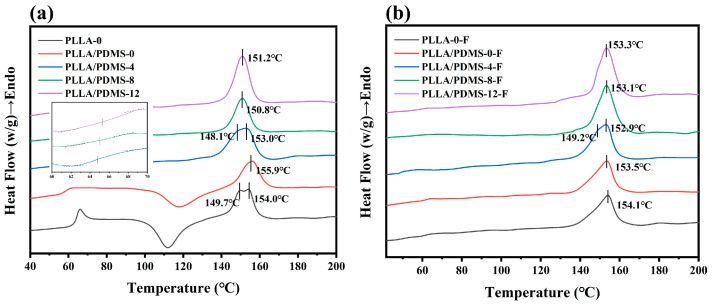
DSC curves for (**a**) before and (**b**) after the samples were foamed with different orientation ratios.

DSC curves are shown in Figure 3. The addition of PDMS increased the mobility of the molecular chain in the system, resulting in an early glass transition, as shown in Figure 3a. In addition, orientation-induced crystallization and a regular molecular chain structure limit the glass transition, making it unobvious in the system. Moreover, the “frozen” molecular chains after orientation made it difficult to rearrange the molecular chains during the cold crystallization process, resulting in the disappearance of the cold crystallization peak. The wide melting peak of PLLA-0 indicated the diversity of its structure, while PLLA/PDMS-4 and PLLA/PDMS-4-F showed a shoulder peak of chain rearrangement. The melting peak of the sample with a high draw ratio was sharper, indicating that the high orientation led to the high regularity of the PLLA structure, which made the melting process clearer [55].

The T_g_, crystallinity (X_c_), melting temperature (T_m_) and lamella thicknesses (ι) of each sample in the heat flow curve are shown in Table 2. Generally speaking, the melting peaks’ motion also corresponds to the change in ι change.

ι is related to the crystallization temperature and processing time [56], and is calculated using the Gibbs–Thomson [57] Equation (6):

(6) $$\iota =\frac{2\sigma_{e}}{{\Delta H}_{f}(1-\frac{T_{m}}{T_{m}^{0}})}$$

where $T_{m}$ is the melting temperature of ι, $T_{m}^{0}$ is the equilibrium melting temperature of the infinite (for PLLA $T_{m}^{0}$ = 207.6 °C), $\sigma_{e}$ is the surface energy of the lamellar folded chain surface of the lamella (for PLLA $\sigma_{e}$ = 6.09 × 10^−2^ J/m^2^), and ${\Delta H}_{f}$ is the melting enthalpy per unit volume of infinite crystal (for PLLA ${\Delta H}_{f}$ = 1.11 × 10^8^ J/m^3^).

As shown in Table 2, after the addition of PDMS, the T_g_ of PLLA decreased from 65.7 °C to 58.4 °C, which also proves that PDMS can enhance the molecular chain activity of PLLA and give the random coil the ability to stretch. On the other hand, the T_g_ of PLLA/PDMS-4 was significantly increased, because the orientation made the molecular chain of PLLA regularly arranged, and with the increase in orientation ratio, the molecular chain became more regular, resulting in the obstruction of intermolecular movement. The glass transition process required more energy, resulting in a higher T_g_. At 400% orientation, melting double peaks appeared due to the imperfect crystal structure, and there were grains of different sizes. The samples had the highest crystallinity, and this was also why PLLA/PDMS-4-F had the highest toughness while maintaining high strength. On the whole, as the degree of orientation increased, crystallinity increased, and the grain size decreased, which can be attributed to the fact that the grains had undergone fragmentation, slip and rearrangement during the high-orientation process, resulting in finer and more regularly arranged grains. Figure S1 and Table S1 show the same results.

### 3.3. SEM and Density

**Figure 4:**
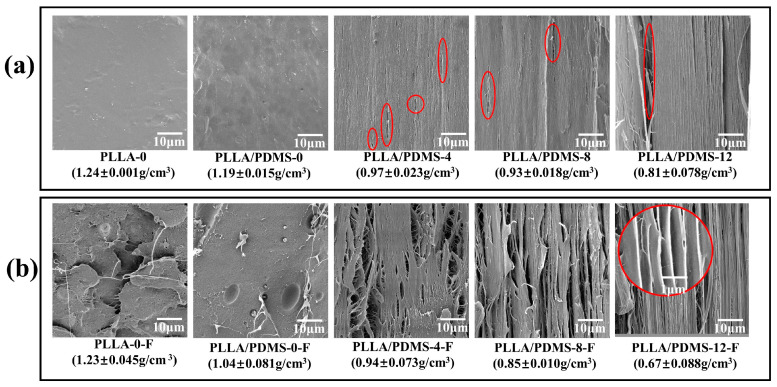
SEM images (10k×) and density (**a**) before and (**b**) after samples were foamed with different orientation ratios.

Figure 4 shows the SEM images and density of the samples. In Figure 4a, showing the results of the high-orientation process, PLLA exhibits a micropore structure along the stretching direction, because stress concentration occurred during stretching, leading to localized plastic shear deformation at stress-concentrated sites. As the localized plastic deformation increased rapidly, a sufficient lateral stress component accumulated within the plastic deformation zone. When the lateral tension increased to a critical value, micropores were induced in the PLLA within the localized plastic deformation zone, as marked by circles in Figure 4a. Subsequently, the system continued to elongate and deform, and the micropores grew and combined with each other, eventually forming crazes. However, due to the interaction between the crazes and shear bands, the micropores did not continue to grow indefinitely. With an increasing orientation ratio, PLLA molecular chains became more oriented and orderly, resulting in a more regular and compact arrangement of micropores. The addition of PDMS reduced the distance between molecular chains, resulting in a denser structure. This further confirmed that PDMS enhanced the activity of PLLA molecular chains and made the molecular chains more flexible in the orientation process, thereby reducing the effect of conditions such as stress concentration and large cracks. As shown in Figure 4b, after foaming without orientation, the system exhibited uneven or even collapse, but with the increase in orientation ratio, especially after the addition of PDMS, the foam micropores became more uniformly distributed. This indicates that PDMS, orientation and foaming jointly regulated the interaction between crazes and shear bands, which increased the toughness of the material it was being enhanced.

With the increase in the orientation ratio, the density of the material showed a downward trend, due to the emergence of micropores. The material density decreased uniformly after foaming, but the density of PLLA-0-F remained basically unchanged, indicating that pure PLLA could not achieve a good foaming effect after SC-CO_2_ foaming, while the addition of PDMS improved the foaming capacity of PLLA and caused PLLA to be lightweight.

**Figure 5:**
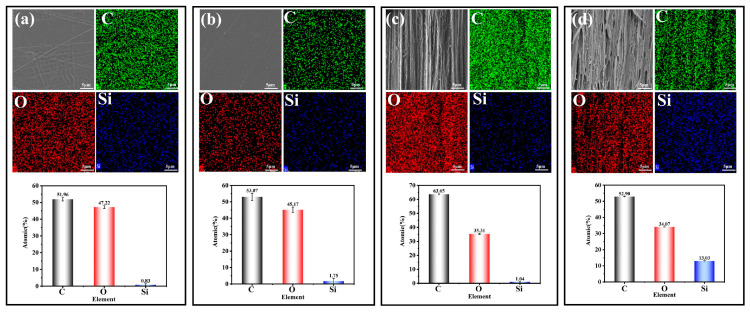
Element distribution on the surface of the samples (10k): (**a**) PLLA-0, (**b**) PLLA-PDMS-0, (**c**) PLLA/PDMS-12, and (**d**) PLLA/PDMS-12-F.

In addition, in order to verify whether there was a phase separation between PDMS and PLLA, which would have affected the uniformity of the material, we determined the element distribution on the surface of the samples. In Figure 5, it can be seen that PDMS was uniformly dispersed in the PLLA matrix, regardless of orientation or foaming, and there was no obvious phase separation.

### 3.4. Strength and Toughness

**Figure 6:**
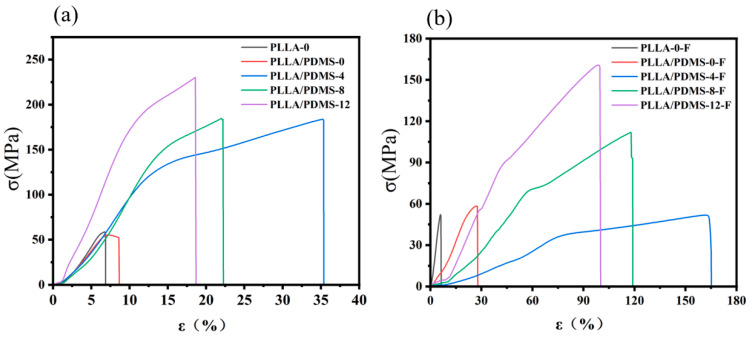
Stress–strain curves of (**a**) un-foamed and (**b**) foamed PLLA samples and with different orientation ratios.

As shown in Figure 6, regardless of whether there was foaming, the tensile strength rose with the increase in the orientation ratio, but the elongation at break decreased. This was due to the molecular chain being stretched and regularly arranged along the orientation direction, as a result of its enhanced ability to resist external force deformation. The change in elongation at break was due to the fact that the molecular chain was stretched and the movement of the molecular chain was limited, resulting in the brittleness of the samples.

After foaming, the tensile strength of the system was greatly reduced and the elongation at break was greatly increased. This was because a large number of microporous structures were formed inside PLLA rather than tightly arranged molecular chains. The presence of lots of microporous structures is more likely to lead to a sharp reduction in tensile strength; even so, the tensile strength of PLLA/PDMS-12-F can still reach 151.2 MPa. On the other hand, the rise in elongation at break was due to the increase in deformable free space remaining in the foamed PLLA.

As shown in Table 3, the modulus of PLLA increased after orientation, which was consistent with the change in strength, indicating that high orientation was beneficial to the regular arrangement of molecular chains to improve crystallinity. The modulus of the foamed samples was greatly reduced, which also showed that the foaming enhanced the ability of PLLA to buffer external forces and that the toughness was greatly improved.

### 3.5. Surface Tension

As shown in Table 4, after the addition of PDMS and under the orientation condition, the samples showed greatly smaller surface tension, and the surface interaction with oil was greatly reduced overall. This indicates that the addition of PDMS and orientation could enhance the hydrophobic and oil-repellent properties of PLLA. This is because PDMS can fill and smoothen the surface microstructure of PLLA, effectively reducing the surface roughness and causing a decrease in the surface tension. The decrease in the surface tension of the material after orientation was due to the fact that the regular molecular chain structure greatly improved the crystallinity of PLLA and made the surface structure more stable.

After foaming, the surface tension and interaction force with oil further decreased. This indicates that SC-CO_2_ foaming could effectively enhance the hydrophobic and oil-repellent properties of PLLA. This was because SC-CO_2_ promoted an increase in system crystallinity, leading to a more ordered arrangement of polymer chains, so it was more difficult for water and EG to penetrate crystalline regions, resulting in a larger contact angle. At the same time, SC-CO_2_ and the microporous structure enhanced the intermolecular interaction forces within the material, while weakening the surface forces, resulting in a decrease in interaction force with oil.

### 3.6. Antibacterial Property

**Figure 7:**
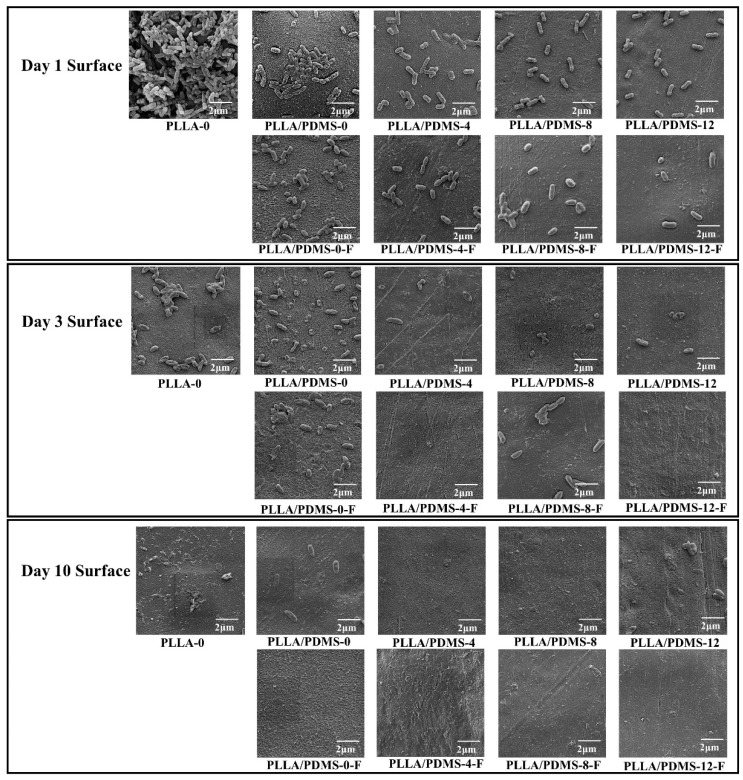
SEM morphology of material surface of samples after 1/3/10 co-culture with Bacillus aeruginosa.

The growth and distribution of Bacillus aeruginosa after a 1/3/10 day co-culture of modified PLLA films are shown in Figure 7. After 1 day of co-culture with the samples, Bacillus aeruginosa prolificated and differentiated on the PLLA-0, stacked thickly on the surface of the film, and the shape was round and full. After the addition of PDMS and the orientation, the amount of Bacillus aeruginosa in the film was greatly reduced, and there was almost no aggregation; most of the bacteria were dispersed, which indicated that the combination of orientation and PDMS had blocked the proliferation and differentiation of Bacillus aeruginosa. Further, the amount of Bacillus aeruginosa in the samples after foaming was reduced, and there was basically no aggregation, which indicates that foaming can hinder the growth of bacteria to a certain extent. On Day 3, the overall amount of Bacillus aeruginosa decreased significantly, as did that on the pure samples (which also had stacking conditions), and were somewhat dispersed on the thin film. It is worth mentioning that on Day 3, some of the Bacillus aeruginosa were no longer full and round, but showed folds and even collapse. All Bacillus aeruginosa showed a flat concave shape, especially after PDMS orientation and foaming. With the extension of co-culture time, by Day 10, all Bacillus aeruginosa were flat and collapsed, especially on the foamed samples, and the amount of Bacillus aeruginosa on the surface of the samples significantly diminished, or there was even almost no Bacillus aeruginosa, indicating that the Bacillus aeruginosa had completely lost the ability for proliferation and differentiation, as well as confirming the long-lasting antibacterial properties of the samples.

**Figure 8:**
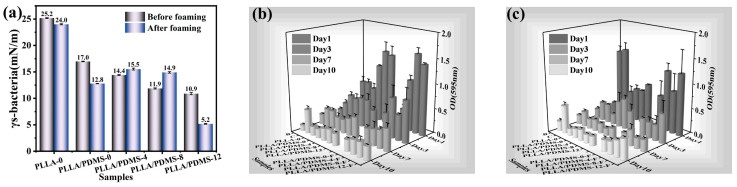
(**a**) Interaction between bacteria and the samples, (**b**) biofilm attachment condition of Staphylococcus aureus, and (**c**) biofilm attachment condition of Bacillus aeruginosa.

In addition to intuitive SEM images, the antibacterial ability of materials can also be analyzed by calculating the interaction force between the samples and bacterial surface to characterize the adhesion ability of bacteria to the samples. The surface force between the samples and bacteria was calculated according to (5), as shown in Figure 8a. The surface interaction between PLLA-0 and bacteria was 25.2 mN/m, and the addition of PDMS also significantly reduced the adhesion ability of bacteria to the film (to 17.0 mN/m). The interaction force between the samples and bacteria gradually decreased with the increase in orientation degree on the whole, indicating that orientation can effectively reduce the adhesion of bacteria. Similarly, SC-CO_2_ foaming also plays a great role in reducing the adhesion of bacteria to a certain extent.

Since Bacillus aeruginosa was a representative of Gram-negative bacteria, Staphylococcus aureus was a representative of Gram-positive bacteria. In order to verify the universality of the antimicrobial properties of the film, Bacillus aeruginosa and Staphylococcus aureus were co-cultured with the samples, and the conditions of their biofilms were measured and expressed by the absorbance value at 595 nm (OD595nm), as shown in Figure 8b,c. As the previous results showed, Staphylococcus aureus and Bacillus aeruginosa showed the same trend: with the extension of culture time, the biofilm content of the two bacteria was significantly reduced and showed a long-term and sustained antibacterial effect.

**Figure 9:**
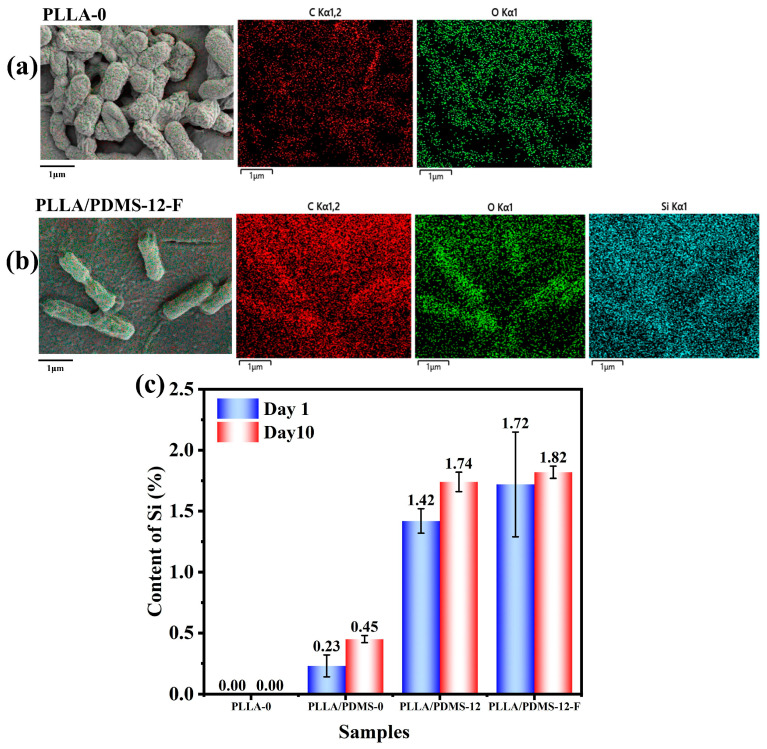
Distribution of elements on the surface of thin film materials, (**a**) PLLA-0 and (**b**) PLLA/PDMS-12-F, and (**c**) the change in Si content.

The above results show that orientation and the addition of PDMS and SC-CO_2_ foam interact with each other to regulate the antibacterial properties of PLLA film materials, achieved a lasting antibacterial effect. In order to explore the bactericidal mechanism of thin film against bacteria, EDS scanning was performed on Day 1 and Day 10 (PLLA-0, PLLA/PDMS-0, PLLA/PDMS-12, PLLA/PDMA-12-F) samples. As shown in Figure 9a,b, after the addition of PDMS, Si (the characteristic element of PDMS) was displayed on the surface of the bacteria to form a layer of PDMS oil film. This PDMS oil film obstructed a series of life activities such as the respiration of bacteria, inhibiting the growth and reproduction of bacteria, so as to achieve an antibacterial effect.

As shown in Figure 9c, the Si content of the samples increased over time after adding PDMS, orienting them, and foaming them. This was because during the orientation process, the enhancement of PLLA molecular chain movement activity led to the PDMS being arranged regularly along the orientation direction, so the PDMS had more active sites in which to interact with PLLA molecular chains, and more PDMS naturally appeared in the system. After foaming, PDMS had more space for movement, so the content increased. As time went on, PDMS surrounded the bacteria and remained on the surface of the material, resulting in accumulation, causing the increase in Si content.

**Figure 10:**
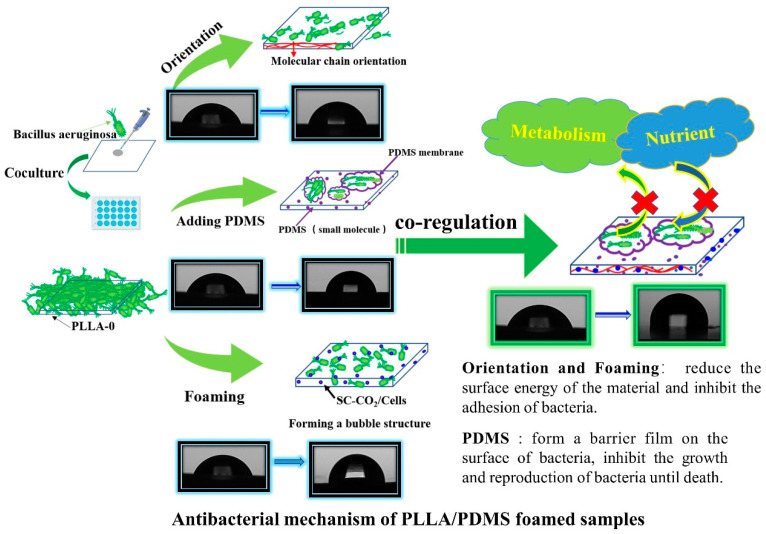
Antibacterial mechanism of PDMS-regulated oriented microporous structure of PLLA.

Based on the above experiments and test results, the antibacterial mechanism was analyzed, as shown in Figure 10. Orientation and SC-CO_2_ foaming mainly played a role in reducing the surface interaction force between the samples and bacteria, thus reducing the adhesion of bacteria to the samples to achieve a antibacterial effect, while PDMS mainly formed an insulating film on the surface of the bacteria, isolated the bacteria from external nutrients, and blocked the growth and reproduction of the bacteria and other life activities from the root. These three phenomena jointly regulated and accurately controlled the adhesion of bacteria to PLLA.

## 4. Conclusions

In this work, PLLA was modified by PDMS, SHD technology and SC-CO_2_ foaming technology. We precisely constructed a PDMS-regulated oriented microporous structure, and realized the high strength, high toughness and lightweight antibacterial properties of PLLA. The results showed that orientation could improve the crystallinity of PLLA to enhance its strength, while SC-CO_2_ foaming could give the molecular chain more movement space to enhance its toughness. Furthermore, the antibacterial mechanism was explored: orientation and foaming could reduce the adhesion of bacteria to achieve an antibacterial effect, while PDMS could block the life activities of bacteria, the three methods jointly regulated the conditions to achieve long-lasting antibacterial properties for PLLA. This work provides an idea for the application of PLLA in packaging materials, especially in food and drug packaging materials with high antibacterial and strength requirements.

## Figures and Tables

**Table 1:** Surface tension of test liquids.

Liquid	$\gamma_{l}$ (mN/m)	$\gamma_{l}^{d}$ (mN/m)	$\gamma_{l}^{p}$ (mN/m)
Distilled water	72.8	21.8	51.0	
Ethylene glycol	48.0	29.0	19.0	

**Table 2:** The thermodynamic parameters for before and after the samples were foamed with different orientation ratios obtained by DSC.

Samples	T_g_ (°C)	ΔH_m_ (J/g)	X_c_ (%)	T_m_ (°C)	ι (Å)
PLLA-0	65.7	36.7	3.0	149.7/154	39.3/42.5
PLLA/PDMS-0	58.4	35.2	5.6	155.9	44.1
PLLA/PDMS-4	64.8	52.5	56.4	148.1/153.0	38.3/41.7
PLLA/PDMS-8	65.0	37.4	40.2	150.8	40.1
PLLA/PDMS-12	65.3	49.0	52.7	151.2	40.4
PLLA-0-F	62.5	40.2	43.2	154.1	42.6
PLLA/PDMS-0-F	61.2	42.3	45.5	153.5	42.1
PLLA/PDMS-4-F	50.9	49.7	53.4	149.2/152.9	39.0/41.6
PLLA/PDMS-8-F	57.6	56.4	60.6	153.1	41.8
PLLA/PDMS-12-F	58.2	57.2	61.5	153.3	42.0

**Table 3:** The tensile strength and Young’s modulus from stress–strain curves.

Samples	Tensile Strength (MPa)	Young’s Modulus (MPa)
PLLA-0	58.6 ± 3.964	1246.2 ± 66.998
PLLA/PDMS-0	59.3 ± 2.040	1046.6 ± 43.061
PLLA/PDMS-4	183.7 ± 5.267	1215.1 ± 21.987
PLLA/PDMS-8	185.7 ± 13.047	1439.6 ± 65.587
PLLA/PDMS-12	230.2 ± 13.702	2333.6 ± 61.658
PLLA-0-F	52.2 ± 4.034	1051.7 ± 67.410
PLLA/PDMS-0-F	58.4 ± 9.814	314.7 ± 14.857
PLLA/PDMS-4-F	51.8 ± 8.061	73.7 ± 1.634
PLLA/PDMS-8-F	111.8 ± 9.087	166.9 ± 9.718
PLLA/PDMS-12-F	151.2 ± 25.973	287.5 ± 2.849

**Table 4:** The surface energy parameter for before and after PLLA samples were foamed with different orientation ratios.

Samples	Contact Angle (°)		γ_s_^d^ (mN/m)	γ_s_^p^ (mN/m)	γ_s_ (mN/m)	γ_s-soybeanoil_ (mN/m)
	Water	EG				
PLLA-0	60.9 ± 0.7	46.3 ± 1.9	13.1 ± 0.016	27.1 ± 0.001	40.2 ± 0.016	21.8 ± 0.017
PLLA/PDMS-0	66.1 ± 0.4	42.5 ± 1.2	17.1 ± 0.006	19.9 ± 0.001	37.0 ± 0.007	14.2 ± 0.006
PLLA/PDMS-4	69.0 ± 0.6	45.1 ± 1.0	18.1 ± 0.001	17.1 ± 0.009	35.2 ± 0.010	11.9 ± 0.002
PLLA/PDMS-8	71.8 ± 0.9	47.2 ± 0.7	19.4 ± 0.001	14.5 ± 0.008	33.9 ± 0.012	9.6 ± 0.010
PLLA/PDMS-12	76.6 ± 1.5	53.5 ± 2.9	18.4 ± 0.027	12.1 ± 0.001	30.5 ± 0.028	8.7 ± 0.028
PLLA-0-F	62.7 ± 0.9	42.7 ± 0.9	13.2 ± 0.001	25.6 ± 0.007	38.8 ± 0.008	20.7 ± 0.007
PLLA/PDMS-0-F	69.1 ± 0.8	43.5 ± 0.6	19.7 ± 0.002	16.1 ± 0.010	35.8 ± 0.013	10.4 ± 0.012
PLLA/PDMS-4-F	71.3 ± 1.5	49.8 ± 1.4	16.1 ± 0.002	16.9 ± 0.022	32.9 ± 0.024	12.9 ± 0.023
PLLA/PDMS-8-F	72.7 ± 1.1	51.4 ± 1.8	16.0 ± 0.004	16.1 ± 0.003	32.1 ± 0.007	12.3 ± 0.007
PLLA/PDMS-12-F	82.6 ± 0.6	55.9 ± 2.2	23.8 ± 0.047	6.6 ± 0.009	30.4 ± 0.056	3.8 ± 0.067

## Data Availability

The supporting data for the study results are available from the corresponding author upon reasonable request.

## Supplementary Materials

The following supporting information can be downloaded at https://www.mdpi.com/article/10.3390/biom14111432/s1: Figure S1: XRD curves of the (a) samples before and (b) after foaming; Table S1: The grain size and crystallinity of the α and β crystal structures.
