# Supplementary material for: Preparation and Antibacterial Properties of Poly (l-Lactic Acid)-Oriented Microporous Materials
Source: Biomolecules. 2024 Nov 11;14(11):1432. doi: 10.3390/biom14111432 (PMC11591793; doi:10.3390/biom14111432)
Supplement: Supplementary file 1 [file biomolecules-14-01432-s001.zip › biomolecules-3249982-supplementary/Supporting information for XRD data and discussion/result and discussion for XRD.pdf]

X-ray diffraction (XRD)

XRD (DX-2500) tests on different materials, at room temperature in a scanning range of 10°-50°, with a scanning rate of 0.03/s and an acceleration current of 20 mA with a copper target (CuKa, λ=0.154 nm).

Based on the XRD diffraction intensity, the sample grain size was calculated using the Scherrer formula:

$$L_{hkl} = \frac{k\lambda}{\beta\cos\theta}$$

where L_{hkl} is the grain size perpendicular to the lattice plane. k is a crystal shape factor, which is generally a constant (related to the definition of β — when β is the full width at half maximum, K is 0.89. When β is the integral width, K is 1.0. λ is the X-ray wavelength (0.154 nm), β is the full width at half maximum of the diffraction peak, and θ is the Bragg diffraction angle(°).

The XRD curve of the samples was fitted using JADE 6.0, while the sample crystallinity (X_c) was calculated using Formula (2):

$$X_c = \frac{A_c}{A_{all}}$$

where A_c is the crystallization peak area, and A_{all} is the total peak area of the XRD curves.

In Figure S-1(a), the molecular chain of the unoriented samples existed in a random coil state, so there was no obvious crystallization signal. However, α and β crystal form gradually appeared after orientation, this proved that orientation makes regular molecular structure. After foaming (Figure S-1(b)), the unoriented samples showed a distinct α (203) crystal plane and a clear β crystal plane. This indicated that foaming further improved the ordered PLLA structure. Additionally, in the foamed samples, the α (203) crystal plane was more prominently observed in the non-oriented state. This was attributed to the fact that in the non-oriented but foamed state, compared with the oriented ("frozen") and foamed state, the addition of SC-CO_2 increased the molecular chain activity of the system, it's easier to form a three-dimensional(3D) orientation structure during foaming, which promoted the

perfection of the α crystal. The β crystal peak intensity increased at a higher orientation ratio, indicating that a high orientation was beneficial for generating a more improved β crystal structure.

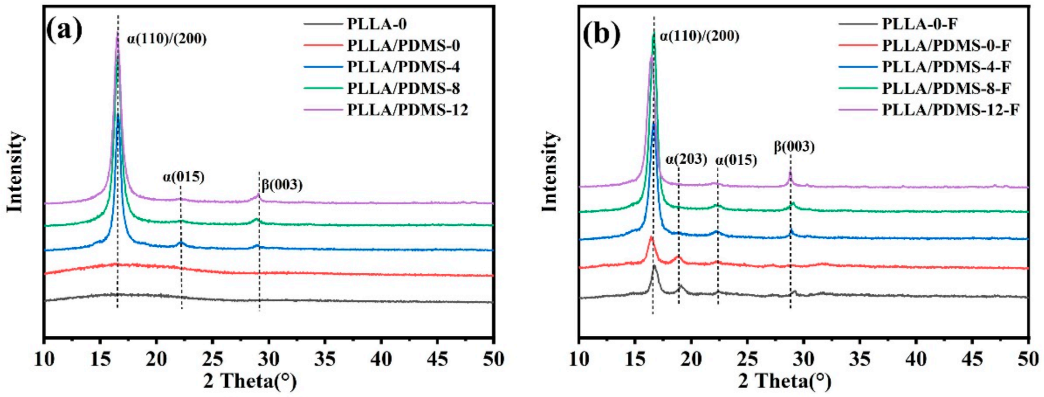

Figure S-1 XRD curves of the (a)samples before and (b)after foaming

As shown in TableS-1, the α (100/200) and β (003) crystal planes displayed the highest diffraction intensity were selected to calculate the crystallinity and grain size. Before foaming, the overall crystallinity increased at a higher degree of orientation, while the grain size decreased. This was because the orientation led to the orderly arrangement of the PLLA molecular chains and slip rearrangement during high orientation to refine the grains. Owing to SC-CO_2 foaming promoted the α and β crystal formation by generating highly stretched micropores in the 3D direction of the PLLA, the crystal grain size was obviously increased.

Table S-1 The grain size and crystallinity of the α and β crystal structures.

Samples	$X_{\alpha\ (110/200)}$ (%)	$X_{\beta\ (003)}$ (%)	X_c (%)	Grain size $_{\alpha\ (110/200)}$ (Å)	Grain size $_{\beta\ (003)}$ (Å)
PLLA-0	—/—	—/—	—/—	—/—	—/—
PLLA/PDMS-0	—/—	—/—	—/—	—/—	—/—
PLLA/PDMS-4	36.1	2.3	41.9	109	119
PLLA/PDMS-8	46.1	2.5	48.3	94	105
PLLA/PDMS-12	53.6	1.8	54.9	105	118
PLLA -0-F	21.1	3.2	27.6	117	123
PLLA/PDMS-0-F	23.7	1.1	28.3	121	133
PLLA/PDMS-4-F	43.9	2.5	46.5	116	167
PLLA/PDMS-8-F	49.8	2.4	53.1	112	120
PLLA/PDMS-12-F	55.7	4.7	68.9	111	185
